# The Chernobyl Nuclear Catastrophe: Unacknowledged Health Detriment

**DOI:** 10.1289/ehp.115-a238

**Published:** 2007-05

**Authors:** Rudi H. Nussbaum

**Affiliations:** Department of Physics and Environmental Sciences, Portland State University, Portland, Oregon, E-mail: D4RN@odin.pdx.edu

Baverstock and Williams (2006) rightly recommended international long-term studies of all potential health effects among the populations exposed to Chernobyl fallout. In the meanwhile, data on post-Chernobyl health detriment in the former Soviet Union and exposed parts of Europe, including evidence of association with such contamination, are already accessible, mostly electronically. Three mutually consistent findings, in particular, challenge widely publicized conclusions the World Health Organization (WHO 2005a, 2005b) (after approval by the International Atomic Energy Agency), and the United Nations Scientific Committee on the Effects of Atomic Radiation (UNSCEAR 2000).

First, scientists from the Moscow Kurchatov Institute presented physical evidence that the dominant sources of energy released by the exploding reactor were not the officially assumed thermal explosions (Fairlie and Sumner 2006) but rather very low-yield nuclear chain reactions in heavy elements, combined with chemical reactions (Checherov 2006). Thus, contrary to the assumed emission of 50 million Ci into the atmosphere (i.e., an estimated 3.5% of the radioactive inventory of the destroyed fuel elements, leaving over 90% of it in the “sarcophagus”), these scientists conclude a 26-fold larger release of radioactivity, leaving no more than 10–15% of the inventory behind. A 26-fold increase would mean that population exposures from the worldwide fallout was in fact more than an order of magnitude larger than assumed by UNSCEAR (2000). This would explain a variety of observed health effects that are not to be expected at currently assumed doses (Committee Examining Radiation Risks of Internal Emitters 2004; Fairlie and Sumner 2006; Glushenko et al. 2006).

Second, the WHO accepted the conclusions by UNSCEAR that exposures of populations in the neighboring contaminated regions were of the order of 10 mSv, except for higher thyroid doses from ^131^I (UNSCEAR 2000; WHO 2005a, 2005b). The main contributions to dose in other tissues—externally and internally—have been assumed to come from ^137^Cs and ^134^Cs, whereas exposures from other radioisotopes, such as ^90^Sr and ^239^Pu, or other alpha emitters were presumed negligible beyond distances of about 100 km from the plant (Fairlie and Sumner 2006; UNSCEAR 2000; WHO 2005a, 2005b).

However, direct biological dosimetry contradicts these official estimates. Several research teams investigated radiation-specific cytogenic alterations in the lymphocytes of about 1,000 exposed persons immediately after the accident and/or some years later (Schmitz-Feuerhake 2006; Schmitz-Feuerhake et al. 2006). The majority of these studies revealed that the rate of unstable and stable chromosome aberrations was about 10–100 times higher than would be expected at UNSCEAR’s estimated exposure levels (UNSCEAR 2000). Biological dosimetry is, however, consistent with the evidence for a much larger release of radioactivity in the explosion. Furthermore, multiaberrant cells, characteristic for incorporated alpha emitters, were identified well beyond 100 km from Chernobyl, whereas plutonium particles were found as far away as Norway, contradicting “negligible exposure levels” beyond 100 km [International Physicians for the Prevention of Nuclear War (IPPNW) 2006; Schmitz-Feuerhake 2006; Schmitz-Feuerhake et al. 2006]. Currently adopted models for Chernobyl dose estimates ignore contributions from alpha emissions even though they are known to have relative biological effectiveness (RBE) about 20 times larger than that of most radioactive beta and gamma radiation (Fairlie and Sumner 2006; International Commission on Radiological Protection 1991; UNSCEAR 2000).

Third, excess infant (perinatal) mortality and teratogenic effects were observed in Germany, Poland, and the former Soviet Union shortly after the Chernobyl explosion [European Committee on Radiation Risk (ECRR) 2006; Gesellschaft für Strahlenschutz/ECRR 2006; Körblein 1997, 2003; Scherb et al. 1999; Schmitz-Feuerhake 2006]. Excess malformations, childhood morbidity, and genetic effects were reported from several areas of Central Europe and Turkey (Committee Examining Radiation Risks of Internal Emitters 2004; ECRR 2006; Fairlie and Sumner 2006; Körblein 2006; Scherb 2006; Schmitz-Feuerhake 2006). These post-Chernobyl observations are consistent with those in the United Kingdom, the United States, and West Germany following the atmospheric nuclear bomb tests of the 1950s (Körblein 2004; Whyte 1992). According to the International Commission on Radiological Protection (1991), UNSCEAR (2000), and other radiation authorities, teratogenic effects should not occur below a dose threshold of about 100 mSv. However, official estimates of fetal doses after the Chernobyl explosion, even in the most contaminated regions of Germany, were < 1 mSv (UNSCEAR 2000), far below the presumed safe threshold. Thus, either the fetus is much more sensitive to radiation than officially assumed, or the estimated post-Chernobyl fetal doses are far too low (which is consistent with considerably higher radioactive releases), or, most likely, there is a combination of both.

In the absence of scientifically convincing evidence rebutting such challenges to official assessments of the physical events and long-term human consequences of the Chernobyl catastrophe, the Precautionary Principle in public health issues (Goldstein 1999; Kriebel et al.2001) requires that these unwelcome findings be no longer ignored in “state of knowledge” reviews (Brenner et al. 2003; National Research Council 2006), in “assessments of the health consequences” (Baverstock and Williams 2006), and in official radiation protection standards.
